# Nicotine Potentially Alters Endothelial Inflammation and Cell Adhesion via *LGALS9*

**DOI:** 10.3390/jcdd11010006

**Published:** 2023-12-23

**Authors:** Sönke Maximilian Braß, Agnesa Mazrekaj, Joscha Mulorz, Wiebke Ibing, Kim-Jürgen Krott, Kiku Takeuchi, Melanie Cappallo, Hsiang-Han Liu, Margitta Elvers, Hubert Schelzig, Markus Udo Wagenhäuser

**Affiliations:** 1 Clinic for Vascular and Endovascular Surgery, Medical Faculty and University Hospital Duesseldorf, Heinrich-Heine-University, 40225 Duesseldorf, Germany; 2 Clinic for Cardiac Surgery, Medical Faculty and University Hospital Duesseldorf, Heinrich-Heine-University, 40225 Duesseldorf, Germany; 3 CURE 3D Lab, Medical Faculty and University Hospital Duesseldorf, Heinrich-Heine-University, 40225 Duesseldorf, Germany

**Keywords:** galectin, nicotine, endothelial cells, aortic pathologies

## Abstract

Background: The endothelial cell layer is essential for the maintenance of various blood vessel functions. Major risk factors for endothelial dysfunction that contribute to aortic pathologies such as abdominal aortic aneurysm (AAA) and aortic dissection (AD) include smoking tobacco cigarettes and hypertension. This study explores the effects of nicotine (Nic) and angiotensin II (Ang II) on human aortic endothelial cells (HAoECs) at a transcriptional level. Methods: HAoECs were exposed to 100 nM Nic and/or 100 nM Ang II. RNA sequencing (RNA-Seq) was performed to identify regulated genes following exposure. Results were validated applying RT-qPCR. GeneMANIA was used to perform in silico analysis aiming to identify potential downstream interacting genes in inflammatory, cell-adhesion, endothelial cell proliferation, and coagulation pathways. Results: RNA-Seq identified *LGALS9* (Galectin-9) as being potentially regulated following Nic exposure, while subsequent RT-qPCR experiments confirmed the transcriptional regulation (*p* < 0.05). Subsequent in silico analysis identified potential candidate genes for interacting with *LGALS9* in different gene sets. Of the top 100 genes potentially interacting with *LGALS9*, 18 were inflammatory response genes, 28 were involved in cell adhesion, 2 in cell proliferation, and 6 in coagulation. Conclusion: Nic exposure of HAoECs causes a significant increase in *LGALS9* at a transcriptional level. *LGALS9* itself may serve as key regulator for essential endothelial cell processes via interfering with various signaling pathways and may thus represent a potentially novel target in the pathogenesis of aortic pathologies.

## 1. Introduction

Vascular endothelium builds the innermost cell layer of the vessel wall and has a critical role in vascular physiology and the preservation of vascular health. It not only serves as a mechanical barrier between the blood stream and the underlying vascular wall but also has endocrine functions [1]. Endothelial cells (ECs) respond to physical and chemical stimuli by releasing factors that regulate coagulation, cell proliferation, cell adhesion, and inflammatory responses [1,2]. However, a variety of risk factors can cause dysfunction of the vascular endothelium, leading to prothrombotic conditions, uncontrolled cell proliferation, overexpression of adhesion molecules, and the generation of a proinflammatory phenotype [3,4,5]. These resulting conditions increase the risk for various atherosclerosis-associated diseases such as abdominal aortic aneurysm (AAA) and aortic dissection (AD) [6,7].

One of the most relevant risk factors for endothelial dysfunction is smoking conventional tobacco and e-cigarettes. Because nicotine (Nic) is one of the main components of e-cigarettes and tobacco products, it remains of utmost interest in current scientific studies. Among the various effects of Nic on the human body, adverse effects on the endothelium have previously been described and play a crucial role in vascular health [8,9,10].

Other than smoking, arterial hypertension (aHT) is another well-established risk factor for the development of cardiovascular disease [11,12]. Of note, aHT is often accompanied by increased plasma levels of angiotensin II (Ang II) [13,14]. Ang II is a vasoconstricting peptide that directly targets the vascular endothelium [15].

The present study aimed to identify candidate genes that are involved in essential endothelial subprocesses such as coagulation, endothelial cell proliferation, cell adhesion, and inflammation and are subject to regulation following Nic and/or Ang II exposure.

## 2. Methods

### 2.1. Cell Culture

Human aortic endothelial cells (HAoECs) (PromoCell, Heidelberg, Germany) were cultivated in Endothelial Cell Growth Medium (PromoCell, Heidelberg, Germany). Human aortic vascular smooth muscle cells (HAoVSMCs) (Sigma-Aldrich, St. Louis, MO, USA) were cultivated in Dulbecco’s Modified Eagle Medium (Thermo Scientific™, Waltham, MA, USA) supplemented with 1% penicillin/streptomycin (Sigma-Aldrich, St. Louis, MO, USA) and 20% fetal calf serum (Biochem GmbH, Berlin, Germany). Cell cultures were grown at 37 °C and 5% CO_2_ (HERAcell240, Heraeus, Hanau, Germany). Light microscopy (Olympus CKX41, Olympus, Tokyo, Japan) was used for morphological cell evaluation. Cells were sub-cultured at 90% confluency using 0.05% trypsin/0.02% ethylenediaminetetraacetic acid (EDTA) (Sigma-Aldrich, St. Louis, MO, USA).

### 2.2. Plate Coating

For RT-PCR validation, Petri dishes were coated with collagen (0.5 mg/mL) (R&D Systems, Minneapolis, MN, USA) or laminin (0.5 mg/mL) (Roche, Mannheim, Germany) before use. Stock solutions were diluted in phosphate-buffered saline (Merck KgaA (Sigma-Aldrich), Darmstadt, Germany) to a final concentration of 0.005 mg/mL for collagen and 0.015 mg/mL for laminin. Petri dishes covered with working solution were placed on a laboratory shaker (MTS 4, IKA^®^—Werke GmbH & Co. KG, Staufen, Germany) for 5 min and kept in an incubator (HERAcell240, Heraeus, Hanau, Germany). After one hour, the working solution was decanted and cells were seeded on coated dishes.

### 2.3. Experimental Conditions

HAoECs (passage 4–10) were treated with 100 nM Nic (Sigma-Aldrich, St. Louis, MO, USA), 100 nM Ang II (Sigma Aldrich, St. Louis, MO, USA), or in a combination of the two (Comb) for 24 h. The chosen concentrations are within the usual range used for in vitro cell culture studies [16,17,18].

### 2.4. RNA Isolation and Complementary DNA Synthesis 

For RNA isolation from cell cultures, RNeasy kits (Qiagen, Hilden, Germany) were used according to the manufacturer’s instructions. RNA was eluted in 30 μL RNase-free water (Qiagen, Hilden, Germany) and was finally quantified using a NanoDrop (NANODROP 2000c Spectrophotometer, Thermo Scientific™, Waltham, MA, USA) at 260 and 280 nm. RNA samples used for whole transcriptome analyses were quantified (Qubit RNA HS Assay, Thermo Fisher Scientific, Waltham, MA, USA) and assessed for quality by capillary electrophoresis using the Fragment Analyzer and the ‘Total RNA Standard Sensitivity Assay’ (Agilent Technologies, Santa Clara, CA, USA). All samples in this study exhibited high levels of RNA quality (RQNs above 9.8).

For complementary DNA (cDNA) synthesis, a high-capacity cDNA Reverse Transcription Kit (Thermo Fisher Scientific, Waltham, MA, USA) was used according to the manufacturer’s instructions with 500 ng of input RNA. The transcription protocol consisted of annealing at 25 °C for 10 min, extension at 37 °C for 120 min, and inactivation of reverse transcriptase at 85 °C for 5 min using a thermocycler (FlexCycler2, Analytic Jena, Jena, Germany).

### 2.5. Library Preparation and Sequencing

Library preparation was performed according to the manufacturer’s protocol using the Illumina Stranded Total RNA Prep, Ligation with Ribo-Zero Plus Library Prep Kit (Illumina Inc., San Diego, CA, USA). Briefly, 500 ng of total RNA were used for ribosomal depletion, fragmentation, the synthesis of cDNA, adapter ligation, and library amplification. Bead-purified libraries were normalized and finally sequenced on a HiSeq 3000/4000 system (Illumina Inc., San Diego, CA, USA) with a read setup of 1 × 151 bp. The bcl2fastq2 tool was used to convert the bcl files to fastq files as well as for adapter trimming and demultiplexing.

### 2.6. Sequencing Data Analysis

Sequencing data were obtained in FASTQ format. Raw reads were mapped to a reference sequence set (hg38) from the USCS genome database using STAR (v2.7.10a) [19,20]. Raw read counts were measured using featureCounts (v2.0.1) [21]. The normalization of raw read counts and testing for differential expression among the various sample conditions were performed using the DESeq2 R package (v1.26.0) [22]. Genes with a maximum 5% false discovery rate (padj ≤ 0.05) were considered to be significantly differentially expressed. The data obtained were displayed in volcano plots created with GraphPad Prism (v10.0.3). Furthermore, the data were subjected to downstream analysis. As such, Gene Set Enrichment Analysis (GSEA) using WebGestalt (v2019) applying log2foldchange gene ranks was performed [23]. The analysis included KEGG pathway and Gene Ontology (GO) analysis.

### 2.7. RT-qPCR

To investigate changes in transcriptional expression, cells were cultured on collagen- or laminin-coated dishes and exposed to the above-mentioned treatments for 6 h. To mimic fluctuating Nic levels in smokers’, exposure to Nic was performed in undulating patterns. Therefore, Nic-containing medium and standard medium were exchanged in 30-min cycles. Next, RNA isolation and cDNA transcription were performed as mentioned above. For mRNA quantification, 1 µL cDNA was added to TaqMan Fast Advanced MasterMix (Thermo Scientific™, Waltham, MA, USA) and specific primers for *LGALS9* (Galectin-9) (HP-101056, Sino Biological, Eschborn, Germany), *LGALS3BP* (Galectin-3-binding protein) (VHPS-5271, Biomol, Hamburg, Germany), and *LGALS3* (Galectin-3) (VHPS-5270, Biomol, Hamburg, Germany). Amplification and fluorescence detection were performed using the CFX96 Real-Time System (Bio-Rad Laboratories, Hercules, CA, USA). Data were normalized to *GAPDH* (330001 PPH00150F, Qiagen, Hilden, Germany) and fold changes were calculated using the ΔΔCt method [24].

### 2.8. In Silico Gene Interaction Analysis

Gene interactions of *LGALS9* were provided by GeneMANIA (v3.6.0) [25]. Overlap statistics were calculated using Venny (v2.1.0) comparing 100 *LGALS9* interaction genes to the inflammatory response (GO:0006954), cell adhesion (GO:0007155), endothelial cell proliferation (GO:0001935) and coagulation (GO:0050817) gene sets from the Molecular Signatures Database (MSigDB) [26,27,28].

### 2.9. Statistics

To detect outliers among the data sets, we utilized Grubbs’ test. Furthermore, we used a one-way ANOVA with the Dunnett correction and *t*-test for statistical analysis. Statistical significance was defined by a *p*-value of ≤0.05. Data were displayed as mean ± standard error of the mean (SEM), with individual data points in bar graphs or dot plots.

## 3. Results

### 3.1. RNA-Sequencing Analysis 

**Figure 1:**
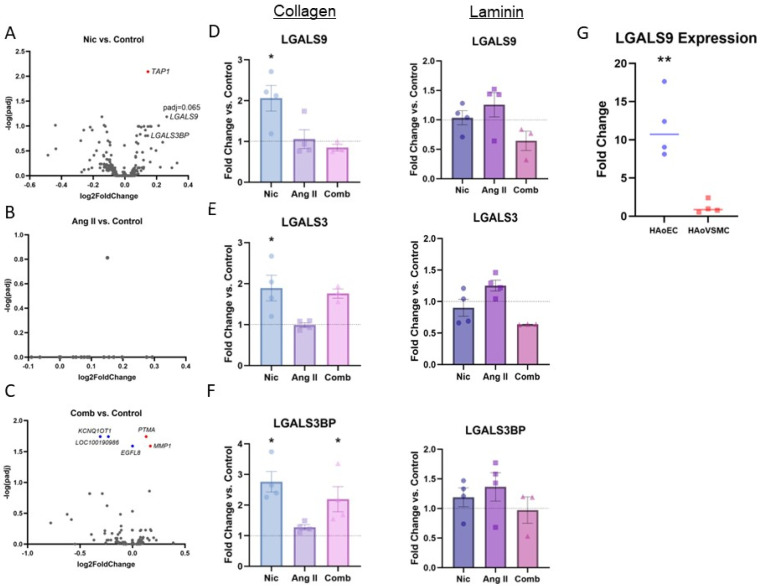
Transcriptomic analysis shows up-regulation of Galectins. (**A**–**C**): Volcano plots show differentially expressed genes (DEG) from RNA-Seq analysis of HAoECs exposed to nicotine (Nic) at 100 nM (**A**), angiotensin II (Ang II) (**B**) at 100 nM and the combination (Comb) of Nic (100 nM) and Ang II (100 nM) (**C**) vs. control. The significant down-regulated genes (blue) and up-regulated genes (red) with padj < 0.05 are highlighted. Relevant genes are labeled; n = 3. (**D**–**F**): Transcriptional levels of relevant genes in HAoECs that were cultured on either collagen- or laminin-coated plates and exposed to Nic, Ang II, and Comb are presented. Transcriptional levels were analyzed applying the ΔΔCT method and normalized vs. *GAPDH*. Data are presented as fold changes vs. control. * *p* < 0.05 vs. control. Grubbs’ test and one-way ANOVA with Dunnett correction were applied; n = 3–4. (**G**): Baseline transcriptional levels of *LGALS9* in HAoECs and HAoVSMCs are presented. Transcriptional levels were analyzed applying the ΔΔCT method and normalized vs. *GAPDH*. Data are presented as fold changes vs. HAoVSMCs. ** *p* < 0.005. Student’s *t*-test was applied; n = 4.

RNA-Seq was performed to identify differentially expressed genes following Nic and/or Ang II exposure in HAoECs. The analysis found *TAP1* (transporter associated with antigen processing 1) as being significantly upregulated following Nic exposure (Figure 1A). *LGALS9* (padj = 0.065) and *LGALS3BP* (padj = 0.16) also showed a clear tendency to upregulate (Figure 1A). Of note, Ang II exposure did not lead to significant transcriptional changes (Figure 1B). When HAoECs were subjected to simultaneous exposure to Nic and Ang II, upregulation of *MMP1* (matrix metalloproteinase 1) and *PTMA* (prothymosin α1) and downregulation of the *KCNQ1OT1* (KCNQ1 opposite strand/antisense transcript 1), *EGFL8* (epidermal growth factor-like protein 8), and long non-coding RNA (lncRNA) *LOC100190986* gene were observed (Figure 1C). The sequencing data were further analyzed applying GSEA including KEGG pathways and GO analysis (Supplementary Figures S1 and S2).

### 3.2. RT-qPCR 

After initial screening for possible regulatory effects, *LGALS9* and *LGALS3* both appeared as potentially relevant regulators of several essential endothelial functions, such as the inflammatory response and cell adhesion. Further RT-qPCR confirmed transcriptional upregulation of *LGALS9* (Figure 1D) and *LGALS3* (Figure 1E) and the associated binding protein *LGALS3BP* (Figure 1F). Of note, such regulation could only be replicated by culturing HAoECs on collagen-coated culture plates (Figure 1D,E). Furthermore, the baseline expression of *LGALS9*, which showed the most robust transcriptional response following Nic exposure, was higher in HAoECs when compared to HAoVSMCs (Figure 1G). 

### 3.3. In Silico Gene Interacton Analysis

**Figure 2:**
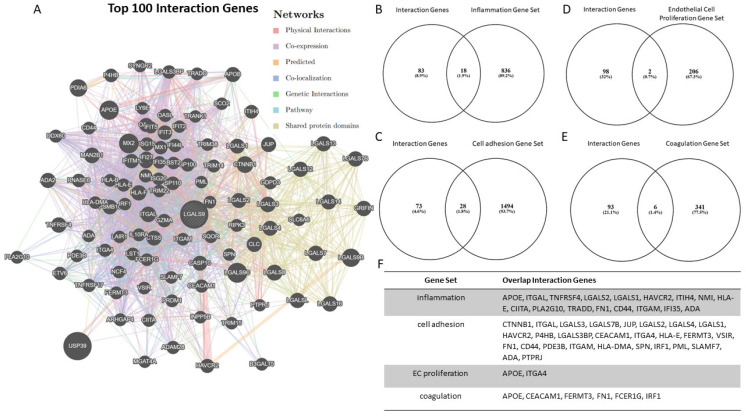
In silico analysis of potential interaction genes for *LGALS9*. (**A**): Gene interaction network shows 100 gene interactions of *LGALS9*. (**B**,**C**): Venn diagrams illustrate the number of overlapping genes between *LGALS9* interaction genes and gene sets for inflammation (**B**), cell adhesion (**C**), endothelial cell proliferation (**D**) and coagulation (**E**). Overlapping genes are listed by GeneMANIA interaction rank order (**F**).

Because *LGALS9* demonstrated the strongest potential for transcriptional regulation in the previous experiments, this gene was further subjected to in-depth in silico interaction analysis. In this regard, its potential for interactions in inflammation, cell adhesion, endothelial cell proliferation, and coagulation was investigated. Of the top 100 genes demonstrating potential interactions with *LGALS9* (Figure 2A), 18 genes are involved in the inflammatory response (Figure 2B), 28 in cell adhesion (Figure 2C), 2 in cell proliferation (Figure 2D), and 6 in coagulation (Figure 2E).

## 4. Discussion

The results of this work comprehensively suggest a significant transcriptional response by galectin genes upon Nic exposure in cultured HAoECs, whereas Ang II exposure did not produce a significant finding. Furthermore, our data show that *LGALS9* is more highly expressed in HAoECs when compared to HAoVSMCs, another primary cell type of the aortic wall. This observation suggests that the endothelial cell layer and its underlying basement membrane are the main sites of action of *LGALS9* within the vasculature. *LGALS9* may regulate various essential endothelial functions by potentially interacting with candidate target genes.

Galectins are a family of proteins with a binding affinity for β-galactoside carbohydrates via a highly conserved carbohydrate recognition domain (CRD) [29]. The prevalent galectin family members in ECs such as HAoECs are galectin-1, -3, -8 and -9 [30]. Of these, *LGALS9* encodes for galectin-9 (GAL-9), a 36 kDa protein containing two N- and C-terminal CRDs connected by a linking peptide, making it a tandem repeat protein [31,32,33].

To date, GAL-9 has been mainly investigated in the context of tumorigenesis, with upregulation of *LGALS9* expression in various cancer types having been reported [34,35]. Nonetheless, the role of GAL-9 in the context of vascular pathologies has been increasingly investigated as recent observational studies have linked elevated serum levels of GAL-9 and other endothelially expressed galectins to vascular diseases such as coronary artery disease (CHD), stroke, and peripheral artery occlusive disease (PAOD) [36,37,38,39]. Although it has been suggested that these elevated plasma levels originate from ECs, the exact pathophysiologic mechanism that contributes to increased endothelial GAL-9 expression remains unclear [40].

Therefore, it seems reasonable to analyze the more precise signaling mechanisms of this protein with regard to its involvement in essential endothelial cellular functions. Our in silico analysis has revealed a high potential for the interaction of *LGALS9* with genes that are involved in the inflammatory response and cell adhesion, in which a large number of the candidate genes appear to be involved. To emphasize this observation, approximately every fourth interacting gene is involved in cell adhesion and every fifth interacting gene is involved in the inflammatory response. Of note, both processes are of utmost interest because they play pivotal roles in the pathophysiology of several atherosclerosis-associated diseases. For instance, AAA formation in this respect is fundamentally based on the surface expression of cellular adhesion proteins, which in turn allow for inflammatory cell transmigration, also called leukocyte trafficking [6,41].

In the context of leukocyte adhesion, GAL-9 may have various functions. Of interest, GAL-9 was initially reported as being involved in the chemoattraction of eosinophils within inflammatory processes [42]. In addition, GAL-9 has recently been introduced as a mediator of neutrophil adhesion, as both in vitro and in vivo experiments have found that GAL-9 leads to increased neutrophil adhesion, which has mainly been linked to the GAL-9–CD44 axis [33]. Of note, our in silico analysis identified *CD44* as one of the major potential interacting genes of *LGALS9* in such a context and therefore supports these previous reports. The expression of CD44 is a suggestive indicator of effector-memory T cells, and one of its major ligands is hyaluronic acid. CD44 is of great interest in vascular inflammation, as investigators have recently demonstrated significant expression levels in atheroma and AAA tissue, suggesting implications for the formation and progression of such diseases [43,44]. Because the aforementioned results are somewhat descriptive, we encourage further experimental studies to obtain better mechanistic insights into the potentially relevant interactions of *LGALS9* and *CD44* in the context of inflammatory vascular diseases such as AAA.

In addition to inflammation, GAL-9 has been reported as being significant in the adhesion of monocytes. Such findings may have implications for atherosclerotic plaque formation, as recently suggested [40]. Pointing in the same direction authors have shown that GAL-9 may increase B cell adhesion in human ECs [45]. Examining this process more closely, such an effect is mainly transmitted by *SLAMF7* (SLAM family member 7). Of note, the *SLAMF7* gene was also found to be one of the most promising interaction candidates for *LGALS9* in our in silico analysis. For the above reasons, it may be worth investigating SLAMF7 further. SLAMF7 is a surface antigen that has been linked to macrophage activity in inflammatory diseases [46]. To the best of our knowledge, SLAMF7 has not been described as involved in inflammatory cardiovascular disease but potentially could be found to play an important role in the future.

Yet, the in silico analysis identified other candidate genes of *LGALS9*-interacting genes that may play pivotal roles in cardiovascular disease. One such gene is *TNFRSF4* (TNF receptor superfamily member 4), which encodes OX40, a member of the TNF receptor family that is capable of recruiting T cells into inflamed tissues via its ligand OX40L [47]. Experimental mouse studies have revealed substantial results, as blocking the OX40/OX40L axis in ApoE-deficient mice led to a 53% reduction in atherosclerotic plaque formation [48]. Another potentially interacting candidate is *ITGAM* (integrin alpha M), which has been described as relevant in the progression of AAA [48]. Applying murine AAA models, *ITGAM* (−/−) mice demonstrated attenuated AAA diameter progression and decreased macrophage infiltration [47]. Notably, disease-relevant interactions between GAL-9 and OX40 and/or ITGAM have not been reported at present, although we believe that addressing interactions in this context could be useful.

In addition to the aforementioned implications, GAL-9 is known for its modulatory role in cell–cell and cell–matrix adhesion [49]. Such a role is supported by the in silico analysis of the present study, which identified *CTNNB1* (β-catenin) and *JUP* (junctional plakoglobin) as potential candidate genes for *LGALS9* interaction. Both CTNNB1 and JUP are known to play critical roles in the cell–cell adhesion of ECs by interacting with VE-cadherin, which is a junctional complexing protein that mediates the mechanical strength of adherent junctions [50]. The role of VE-cadherin in AD has recently been suggested by our group [17]. Toward a mechanistic understanding of the development of AD, experimental research approaches focusing on elucidating the exact roles of CTNNB1 and/or JUP in endothelial cell adherence, barrier function, and permeability, which may generate valuable results in the future.

The present study has various limitations. First, the true environmental conditions in the human body can only be partially mimicked by experimental in vitro conditions. Because tobacco cigarettes contain not only Nic but also numerous other substances that may have effects on the body, this study cannot provide any clear conclusions regarding actual pathophysiological correlations. Therefore, the use of Nic and Ang II to mimic the smoking of tobacco cigarettes or hypertension may be considered only partially valid. This condition also indicated that changing plasma levels of these substances over the course of a day cannot be adequately simulated. In addition, these experiments were performed under static culture conditions, although dynamic conditions may be capable of altering *LGALS9* expression levels as well [39,51].

In conclusion, *LGALS9* appears to be a promising candidate in the Nic-based transcriptional response in HAoECs, and in turn may alter endothelial functions, including cell adhesion and inflammation. Our results encourage further in-depth analyses of the possible interactions of *LGALS9* as well as studies of their implications in signalling cascades, especially in the pathogenesis of cardiovascular disease.

## Data Availability

The underlying data are available from the corresponding author upon reasonable request.

## Supplementary Materials

The following supporting information can be downloaded at: https://www.mdpi.com/article/10.3390/jcdd11010006/s1, Figure S1: KEGG Pathway Gene Set Enrichment Analysis; Figure S2: Gene Ontology Analysis.
