# Supplementary material for: Nicotine Potentially Alters Endothelial Inflammation and Cell Adhesion via LGALS9
Source: J Cardiovasc Dev Dis. 2023 Dec 23;11(1):6. doi: 10.3390/jcdd11010006 (PMC10816207; doi:10.3390/jcdd11010006)
Supplement: Supplementary file 1 [file jcdd-11-00006-s001.zip › jcdd-2757103-supplementary.pdf]

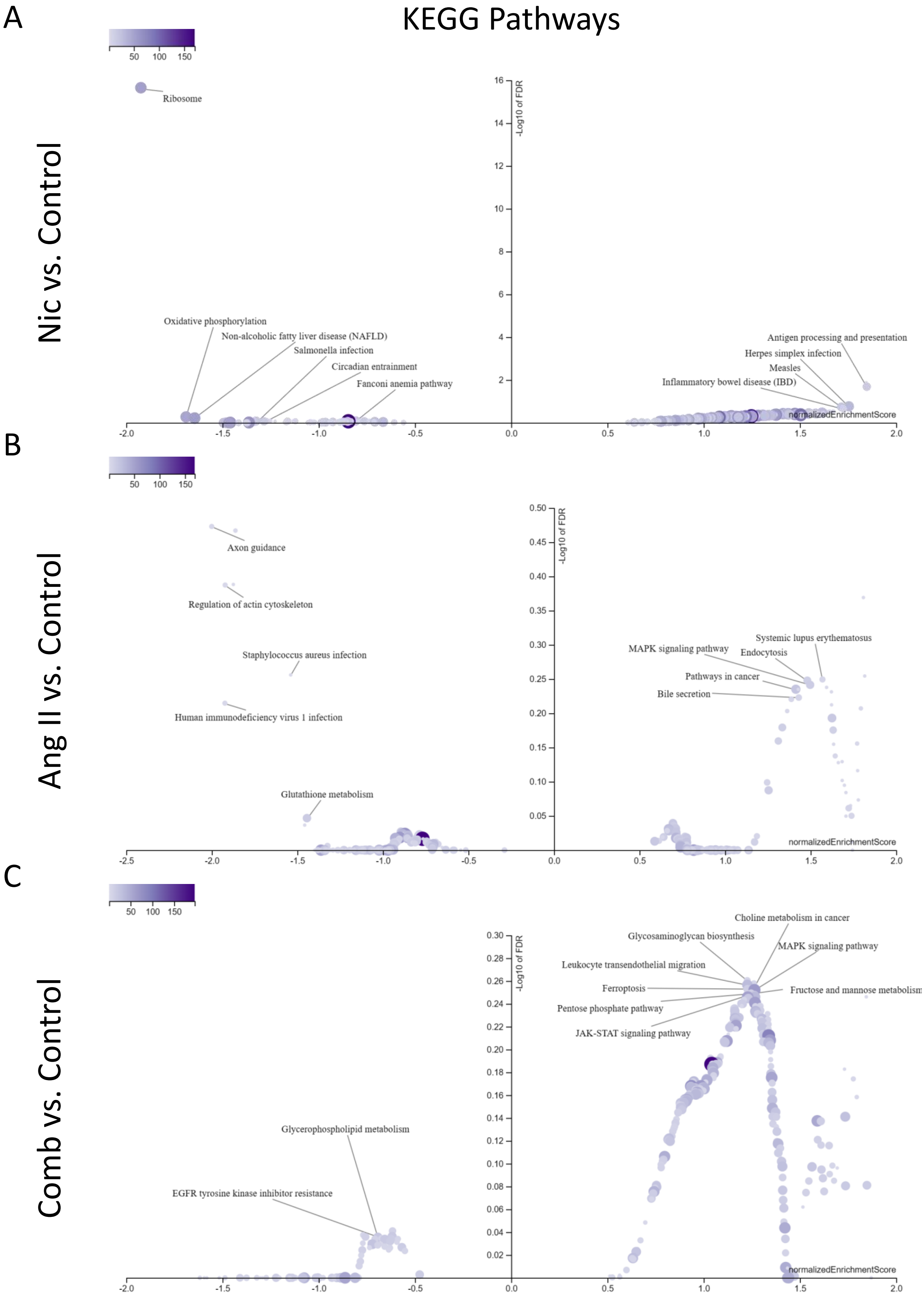

Supplementary Figure S1. KEGG Pathway Gene Set Enrichment Analysis. A-C: Volcano plots show enriched KEGG pathways from RNA-Seq analysis of HAoECs exposed to nicotine (Nic) at 100 nM **(A),** angiotensin II (Ang II) **(B)** at 100 nM and the combination (Comb) of Nic (100 nM) and Ang II (100 nM) **(C)** vs. Control.

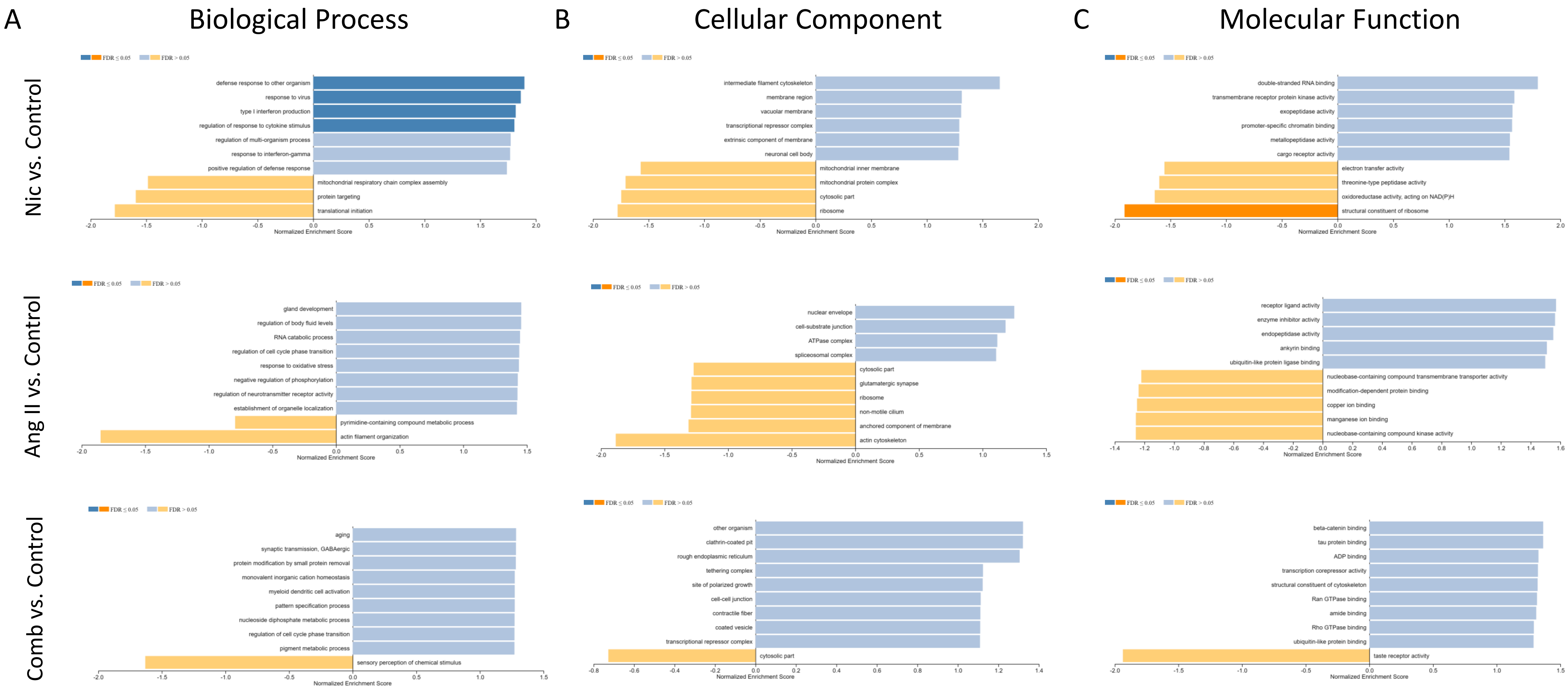

Supplementary Figure S2. Gene Ontology Analysis. A-C: Bar graphs show overrepresented gene sets in biological processes **(A)**, cellular components **(B)**, and molecular functions **(C)** from RNA-Seq analysis of HAoECs exposed to nicotine (Nic) at 100 nM, angiotensin II (Ang II) at 100 nM, and the combination (Comb) of Nic (100 nM) and Ang II (100 nM) vs. Control.
